# Pedicled Supraclavicular Artery Island Flap Versus Free Radial Forearm Flap: Perioperative Outcomes in Head and Neck Reconstruction

**DOI:** 10.7759/cureus.13213

**Published:** 2021-02-08

**Authors:** Jose A González-García, Carlos M Chiesa Estomba, Jon A Sistiaga-Suarez, Ekhiñe Larruscain, Juan D Urazan-Murcia, Xabier Altuna

**Affiliations:** 1 ENT - Head and Neck Surgery, Hospital Universitario Donostia, San Sebastian, ESP; 2 ENT - Head and Neck Surgery, Hospital Universitario Donostia, Donostia, ESP

**Keywords:** free tissue flap, pedicled flap, head, neck, reconstruction

## Abstract

Introduction: Radial forearm free flap (RFFF) and supraclavicular artery island flap (SCAIF) are some of the most common fasciocutaneous flaps used for head and neck (H&N) reconstruction.

Material and methods: A retrospective analysis of clinical data and outcomes of 31 consecutive patients who underwent H&N reconstruction using either SCAIF or RFFF over a three-year period, aiming to compare the surgical outcomes of the SCAIF and the RFFF in H&N reconstruction.

Results: Thirty-two flaps were performed in 31 patients (17 SCAIFs and 15 RFFFs). There was no difference in patient demographics between both groups. Hospital stay was longer in the SCAIF group (30.7 ± 18.2 days (min: 9/max: 60) versus 19.2 ± 15.8 days (min: 7/max: 72). Patients who underwent reconstruction with a SCAIF had shorter reconstructive procedure time; 74.4 min (min: 60/max: 93) versus 147.8 min (min: 140/max: 187). Overall morbidity was not significantly different (SCAIF 52.7% vs RFFF 39.9%, p = NS). Global flap survival was higher without statistical significance in the RFFF group (100%) versus the SCAIF group (70.7%).

Conclusion: Despite the advantages related to the use of SCAIF like regarding the time spent in the reconstructive procedure. In our experience, the RFFF continues to be the most successful technique with similar perioperative outcomes and fewer complication rates. In this vein, both techniques can be reasonably used to reconstruct post-ablative H&N defects. However, in our experience, the use of SCAIF may lengthen hospital length of stay probably due to the augmented risk of flap failure.

## Introduction

Head and neck (H&N) reconstructive surgery aims to solve those problems occurring post tumor ablative resection from an anatomical region with multiple implications over the patient’s quality of life. Essential functions like speech, mastication, swallowing, or breathing can be affected due to these ablative procedures. Also, the aesthetic defects related to the surgery can be sometimes unacceptable. In this vein, the ideal flap for H&N reconstruction procedures should appropriately restore both form and function of the defect and in just one surgical act.

Free flaps are considered as the first option for complex or tridimensional soft tissue reconstruction in the H&N area, mainly the radial forearm free flap (RFFF) and the anterolateral thigh flap, both considered the workhorse flaps by H&N reconstructive surgeons [1-5]. However, some patients may not be suitable for free tissue transfer (FTT) because of older age, poor nutritional status, medical comorbidities, vascular problems often associated with high tobacco or alcohol consumption, the higher risk of prolonged surgeries [6], or the lack of recipient’s vessels because of previous treatments, especially in patients with metachronous or recurrent tumors [7]. Moreover, FTT requires considerable technical expertise, a longer learning curve, special instrumentation, specialized post-operative monitoring and care, and prolonged hospital stay.

In the last years, there is a resurgence of interest in the use of pedicled flaps in H&N. The pectoralis major flap and previously the deltopectoral flap were considered the workhorse flap in H&N reconstruction historically. However, its thickness, limited length, and bulky pedicle [8] were considered the main drawback of this flap that supports the increasing use of the supraclavicular artery island flap (SCAIF) as a proper fasciocutaneous pedicled flap. The SCAIF represents an easy to harvest flap composed of a thin and pliable skin paddle. Due to flap versatility, it is useful for soft tissue defects in the facial, temporal, or parotid region, and upper aerodigestive reconstruction including the oral cavity, pharynx, or larynx [9-15]. The SCAIF elevation leaves negligible morbidity in the donor site with minimal postoperative care needs, making it an effective option for complex defect coverage in the H&N area [16,17].

Literature comparing both techniques is scarce. However, some aspects have been considered previously, like short-term postoperative outcomes demonstrating that both options are reasonable for defects that can be reached by the SCAIF pedicle, with a little advantage in favor of the SCAIF [18-21].

This work aims to evaluate surgical outcomes between two groups of consecutive patients needing reconstruction with either a SCAIF or an RFFF in terms of clinical and demographic data, length of stay, operative time, and flap-related complications.

## Materials and methods

After the approval of the Ethics Committee of Donostia University Hospital, thirty-one patients (21 men and 10 women) who suffered from a primary H&N cancer, and underwent soft tissue reconstruction with either SCAIF or RFFF between January 2017 and January 2020, were evaluated retrospectively. All patients were evaluated in the H&N cancer multidisciplinary committee, and reconstruction was chosen depending on tumor and patient characteristics. All flaps were elevated by the first two authors. Both reconstructive surgical techniques were performed following the standard techniques. Patients younger than 18 years, those who need a composite reconstruction, or those when other types of flaps were used were not included. 

Demographic and clinical data were collected and compared between both groups. Age, sex, comorbidities, alcohol and tobacco consumption, hospital stay, flap size, reconstructive surgical time, type of surgery, TNM stage (seventh edition), histology, and tumor differentiation data were extracted from the medical records. Also, specific flap-related complications were investigated. Total reconstructive procedure time includes flap elevation, insetting, and in the case of an RFFF, the time spent in the microsurgical venous and arterial anastomosis. In RFFF reconstructions, only one vein anastomosis, including the superficial and deep venous systems was performed. In five cases, the SCAIF was used as a back-up flap, while just in one case the RFFF was used as a back-up flap. In our institution, all FTT patients spend 24 hours in an intermediate care unit.

Descriptive results were expressed as mean ± standard deviation. The Shapiro-Wilk test was used to assess the normal distribution between both groups. For dichotomous variables, Fisher’s exact test and Pearson’s Chi-square test were used. A p-value <0.05 was considered significant. Because the main target of this study was to analyze both reconstructive techniques, Student’s t-test was used for quantitative variables. The confidence interval (95%) was calculated. Regarding surgical time, the authors just compare the time spent during the reconstructive procedure and not the total surgical time.

## Results

Patient’s demographic data (medical background, alcohol and tobacco consumption, hospital length of stay, mean reconstructive surgical time, type of surgery, TNM stage, and histologic findings) are presented in Table 1.

**Table 1:** Demographic and clinical data analysis. SCAIF: supraclavicular artery island flap, RFFF: Radial forearm free flap, DM: diabetes mellitus, AHT: arterial hypertension, COPD: chronic obstructive pulmonary disease, SCC: squamous cell carcinoma, ACC: adenoid cystic carcinoma.

Variable	SCAIF	%	RFFF	%	p-Value
Sex					
Male	11	68.8	10	66.7	0.001
Female	5	31.3	5	33.3	
Flaps	17	100	15	100	
Main flap	12	70.5	14	93.3	
Back-up flap	5	29.5	1	6.7	
Mean age	71 ± 12 years (min: 50/max: 91)		56 ± 10 years (min: 49/max: 72)		0.133
Comorbidities					
DM	6	37.5	1	6.7	0.125
HTA	8	50	5	33.3	0.129
BMI <18.5	3	18.8	1	6.7	0.101
Obesity	5	31.3	3	20	0.12
COPD	3	18.8	2	13.3	0.101
Heart disease	8	50	2	13.3	0.129
Smoker					0.209
<20 packs per year	4	25	2	13.3	
>20 packs per year	7	43.8	9	60	
No	5	31.3	4	26.7	
Alcohol intake					0.128
<70 g per day	10	62.5	7	46.7	
>70 g per day	6	37.5	8	53.3	
Mean hospital stay	31 ± 18 days (min: 9/max: 60)		19 ± 16 days (min: 7/max: 72)		0.001
Type of surgery					0.062
Total laryngectomy	3	18.8	3	20	
Oropharyngectomy	1	6.3	2	13.3	
Salivary gland tumor (parotid)	1	6.3	0	0	
Oral cavity	4	25	9	60	
Skin cancer temporal bone and zygoma	2	12.5	0	0	
Cutaneous defect	5	31.3	0	0	
Skin cancer—midfacial resection	0	0	1	6.7	
T stage					0.705
T2	2	12.5	3	20	
T3	2	12.5	3	20	
T4a	12	75	9	60	
N stage					0.724
N0	9	56.3	8	53.3	
N1	3	18.8	1	6.7	
N2a	0	0	0	0	
N2b	2	12.5	0	0	
N2c	0	0	3	20	
N3a	1	6.3	0	0	
N3b	0	0	3	20	
M stage					1
M0	16	100	15	100	
Histology					1
SCC	15	93.8	15	100	
ACC	1	6.3			
Differentiation grade (SCC)					0.162
Well	4	25	1	6.7	
Moderately	8	80	11	73.3	
Poorly	4	25	3	20	

The distribution between both groups was normal according to the Shapiro-Wilk test (p=0.130). Thirty-two flaps were elevated in 31 patients (one patient needed two SCAIF). The SCAIF was used in 16 patients (11 men and five women), and the RFFF was used in 15 patients (10 men and five women). The need for fasciocutaneous flaps was more frequent in men (Fisher - p=0.001). We also tend to use the SCAIF in older patients, 70.6 ± 11.8 years (min: 50/max: 91), while the RFFF is used in younger patients, 56.1 ± 10.3 years (min: 49/max: 72; Student T-test - p=0.130). The overall mean area for the SCAIF donor site was 61.72 cm^2^ (min: 36 cm^2^/max: 64 cm^2^), compared with 78.2 cm^2^ (min: 36 cm^2^/max: 98 cm^2^) for the RFFF group (p = 0.056). The donor site defect was not quantified.

**Table 2:** Flap-related complications. SCAIF: supraclavicular artery island flap, RFFF: radial forearm free flap.

Flap-related complications	SCAIF	%	RFFF	%	p-Value
No	8	47	9	60	0.502
Recipient site dehiscence	1	5.8	2	13.3	
Donor site dehiscence	3	17.6	4	26.6	
Partial flap necrosis	3	17.6	0	0	
Total flap necrosis	2	11.7	0	0	
Total	17	10	15	100	

We found a significant difference between both groups according to the mean length of hospital stay; the RFFF group was discharged in 19 ± 16 days (min: 7/max: 72) while the SCAIF group was discharged in 31 ± 18 days (min: 9/max: 60; p=0.001; Table 2). Regarding surgical time, it was significantly lower in the SCAIF group (74.4 min = min: 60/max: 93) compared to the RFFF group (148 min = Min: 140/Max: 187; p=0.001). While the meantime to elevate an RFFF was 76 min (min: 61/max: 95) and the meantime to elevate the SCAIF was 45 min (min: 31/max: 66), being those differences statistically significant (p=0.001; Table 3).

**Table 3:** Comparison of time specifically spent in the reconstructive procedure in each group. SCAIF: supraclavicular artery island flap, RFFF: radial forearm free flap, CI: confidence interval.

Variables	RFFF	SCAIF	p-Value	CI
Elevation	76 min (min: 61/max: 95)	45 min (min: 31/max: 66)	0.001	30.9 (95% = 23.3 to 38.7)
Insetting	28 min (min: 19/max: 37)	30 min (min: 25/max: 41)	0.397	(−) 1.63 (95% = −5.51 to 2.25)
Flap overall mean area	78.2 cm^2^ (min: 36 cm^2^/max: 98 cm^2^)	61.72 cm^2^ (min: 36 cm^2^/max: 64 cm^2^)	0.056	N/A
Arterial anastomosis	23.9 min (min: 21/max: 36)	N/A		
Venous anastomosis	28.6 min (min: 25/max: 37)	N/A		
Total	148 min (min: 140/max: 187)	74 min (min: 60/max: 93)	0.001	83.85 (95% = 74.5 to 93.19)

## Discussion

Head and neck cancer surgery represents a mutilating procedure requiring, in some cases, the use of reconstructive procedures to assure aesthetic and functional outcomes. Although the use of FTT is still considered the workhorse in H&N reconstruction, pedicled flaps may be regarded as an excellent option when FTT is not indicated.

According to our findings, we demonstrated similar results between the use of both flaps in H&N reconstruction, with the exception that those patients reconstructed with an RFFF were discharged earlier from the hospital, and the reconstructive procedure was shorter in the SCAIF group. Moreover, the elevation time does not affect total procedure time (resection + reconstruction) when, as in our case, the surgery is performed in a two-team approach. However, some authors advocate that when a SCAIF is used for H&N reconstruction, flap elevation is challenging during the surgical resection even working as a two-team approach. Although when the contralateral neck is being dissected, the elevation of a SCAIF is still feasible, improving the total surgical time [18].

For experienced reconstructive surgeons, surgical time does not differ when performing an RFFF or a SCAIF [19], and the time needed to achieve an optimal result can be reduced with a growing surgeon´s experience. As Sukato et al. demonstrated in a recently published systematic review of four studies comparing the use of the RFFF versus SCAIF in H&N reconstruction [22]. In this review, surgical times for SCAIF and FTT cohorts were 368.68 (105.4) minutes and 462.1 (113.9) minutes, respectively, with a large effect size in favor of SCAIF [22].

When comparing hospital length, previous authors have reported no differences between SCAIF and RFFF groups [19] or reduced hospital stays in the SCAIF group [18]. However, in our experience, patients reconstructed with an RFFF are discharged earlier from the hospital. Being necessary to highlight that the nature of the hospital stays in both cases is different, because as we mention above, in our institution, those patients who undergo an FTT spend 24 hours in the ICU.

About those factors that can be related to the shorter length of stay, the absence of vascular complications in patients reconstructed with an RFFF in our study can be one of the main reasons. By contrast, five patients needed additional surgeries in the SCAIF group due to partial or total flap necrosis, consequently incrementing the total length of hospital stay. Remarkably, our first five cases of H&N reconstruction with a SCAIF were all successful [17] and without complications. Nevertheless, such good results might be also due to the small study sample and complications can be expected when incrementing the number of patients. These complications could be related to older age or comorbidities, as this group tends to include patients not suitable for FTT, with a negative selection bias. As prior authors have been remarked, the SCAIF improved surgical efficiency, because just one surgeon can be enough to elevate the flap, minimizing the use of operating room personnel requirements [19].

Regarding surgical complications, we could not find any statistical difference among both groups, despite the absence of complications in those patients treated with an RFFF, compared to the total loss of two flaps and the development of three partial flap necrosis in the SCAIF group. Also, Granzow et al. [18], Kozin et al. [19], and Welz et al. [20] did not find statistical differences regarding complications. However, Zhang et al. [21] reported a significant increase in donor-site complications in the RFFF group, mainly hypertrophic scarring and partial skin graft necrosis without differences in recipient-site complications. When systematically compared, it seems that SCAIF prevents total flap loss while RFFF prevents partial flap loss without statistical significance. Also, RFFF use appears to increase donor-site dehiscence, and SCAIF seems to increase recipient-site dehiscence, although differences could have been missed because of short samples reducing statistical power [22].

The addition of pedicled SCAIFs for H&N reconstruction can significantly reduce operative times and implies less surveillance of flap vascular status in the first days after surgery, with the extra benefit of reduced postoperative intensive care length of stay as described by the previous authors [18]. Another advantage of using the SCAIF flap as a fasciocutaneous alternative to the RFFF in patients not suitable for FTT or by-election of the surgical team is that hospital costs can be reduced as FTT increases costs when compared in global or by type of oncologic resection [19,20]. Regarding functional outcomes, Zhang et al. [21] reported similar outcomes in the quality of speech and swallowing, when performing hemyglossectomy reconstruction. Still, functional results in different H&N subsites and quality of life after surgery protocols should be obtained in the future to disclose more accurate results.

The main limitations of our study, as in previous similar reports, can be related to the small sample size, making a challenge to establish proper comparison among our results (hospital stay, complication rates, quality of life, etc.), the difference according to each anatomical subsite treated (larynx, oropharynx, salivary glands, etc.), the risk of selection bias, the lack of cost analysis and the lack of a proper functional outcome evaluation. Moreover, the fact that measures of wound healing are inherently subjective and may vary according to each surgeon makes it challenging to establish a rigorous comparison. In this way, we decide to continue with our prospective data collection to re-evaluate our results in the future.

## Conclusions

Despite those well-known advantages related to the use of the SCAIF regarding the time spent during the reconstructive procedure or the cost-effectiveness, in our experience, the RFFF continues to be the most successful technique with similar perioperative outcomes and fewer complication rates. In this vein, both techniques can be reasonably used to reconstruct post ablative H&N defects. However, in our experience, the use of SCAIF may enlarge hospital length of stay probably due to augmented risk of flap failure.
